# Differentiating Degenerative from Vascular Dementia with the Help of Optical Coherence Tomography Angiography Biomarkers

**DOI:** 10.3390/healthcare10030539

**Published:** 2022-03-15

**Authors:** Efthymios Chalkias, Ioannis-Nikolaos Chalkias, Christos Bakirtzis, Lambros Messinis, Grigorios Nasios, Panagiotis Ioannidis, Demetrios Pirounides

**Affiliations:** 1 Department of Ophthalmology, AHEPA University Hospital, GR 54621 Thessaloniki, Greece; timoshalkias@yahoo.com (E.C.); john_halkias@hotmail.com (I.-N.C.); dimpero@gmail.com (D.P.); 2 B’ Department of Neurology, School of Medicine, Aristotle University of Thessaloniki, GR 54636 Thessaloniki, Greece; ioannidispanosgr@yahoo.gr; 3 Lab of Cognitive Neuroscience, School of Psychology, Aristotle University of Thessaloniki, GR 54124 Thessaloniki, Greece; lmessinis@psy.auth.gr; 4 Department of Speech and Language Therapy, School of Health Sciences, University of Ioannina, GR 45500 Ioannina, Greece; grigoriosnasios@gmail.com

**Keywords:** Alzheimer’s, biomarkers, neurodegeneration, small vessel disease, vascular cognitive impairment

## Abstract

Alzheimer’s disease and vascular dementia account for the majority of cases of cognitive decline in elderly people. These two main forms of dementia, under which various subtypes fall, are often overlapping and, in some cases, definitive diagnosis may only be possible post-mortem. This has implications for the quality of care and the design of individualized interventions for these patients. Optical coherence tomography angiography (OCTA) is a non-invasive imaging modality used to visualize the retinal layers and vessels which shows encouraging results in the study of various neurological conditions, including dementia. This review aims to succinctly sum up the present state of knowledge and provide critical insight into emerging patterns of OCTA biomarker values in Alzheimer’s disease and vascular dementia. According to the current literature, vessel density seems to be a common biomarker for both forms; inner retinal layer thickness might represent a biomarker preferentially affected in degenerative dementia including Alzheimer’s, while, in contrast, the outer-layer thickness as a whole justifies attention as a potential vascular dementia biomarker. Radial peripapillary capillary density should also be further studied as a biomarker specifically linked to vascular dementia.

## 1. Introduction

Degenerative dementia, largely represented by Alzheimer’s disease (AD) and vascular dementia (VaD), is an umbrella term for the two main forms of the disease accounting for the vast majority of dementia cases, which are constantly on the rise [1]. Despite their distinct underlying pathophysiological mechanisms, there is a non-negligible overlap that makes differential diagnosis, and thus prompt and meaningful intervention, challenging. The identification of pathological proteins such as Amyloid beta (Aβ) and tau (τ), as well as sensitive and specific magnetic resonance imaging (MRI) findings, to support either diagnosis may require invasive, expensive, and time-consuming means, which are less applicable in a day-to-day clinical setting.

Optical coherence tomography angiography (OCTA) is a non-invasive technology which expands the high-resolution imaging capabilities of the pre-existing technology of structural OCT by adding the possibility of retrieving quantifiable and reproducible data on retinal microvasculature, in addition to its anatomical characteristics. Its wide and eminent applications in ophthalmology became evident almost instantly, bridging the information provided by fluorescein angiography and OCT. However, the technology is quickly, and equally, proving its value in the research of various neurological and neuro-ophthalmic conditions, including multiple sclerosis, optic neuritis and dementia [2].

In this review, we aim to examine available data on OCTA biomarker values in people with a diagnosis falling under either the degenerative or vascular dementia varieties, recognize emerging patterns and determine possible areas of interest for further research. The results of this review may aid the work and planning processes of fellow researchers in this area. Although we do not aim to or claim to present an exhaustive analysis of the existing literature, meticulous attention has been directed towards ensuring an accurate and unbiased exposition of the consensus in the matters studied so that this short review is representative of the current knowledge and understanding in the field.

## 2. OCTA in Alzheimer’s Disease

Alzheimer’s disease (AD) is a neurodegenerative disorder and one of the leading causes of dementia worldwide. It has huge implications for the quality of life of affected individuals, as it interferes with cognitive abilities, reasoning skills, language, and attention and ultimately alters their behavior and personality. In severe cases, it can lead to death. The pathophysiological mechanism underlying AD involves the accumulation of extracellular Aβ plaques and intracellular τ-proteins in cortical and limbic cerebral areas [3]. The definitive confirmation of AD can only be achieved postmortem by identifying neurofibrillary tangles and Aβ plaques in these parts of the brain [4].

Although OCTA is a relatively new imaging modality and thus its application in dementia is still being explored, the general consensus seems to be that patients with AD show an increase in the foveal avascular zone (FAZ), as well as a decrease in the superficial parafoveal and in the whole-vessel density (VD) [5]. More specifically, in one of the earliest studies on this topic, Jiang et al. showed that AD patients had significantly reduced VD in both the superficial (SCP) and deep capillary plexus (DCP) compared to controls [6]. These results have been replicated in subsequent studies [7,8]. The assessment of the FAZ has also shown some promising results as a potential biomarker for AD, with research suggesting an enlargement of this area in affected individuals [7,9]. The proposed pathological mechanism which might explain these findings is the accumulation of Aβ plaques in the retina in a manner similar to processes affecting the brain. More specifically, it seems that the retinal hypoxia is a combination of the binding of the vascular endothelial growth factor (VEGF) to Aβ and its confinement in the plaques, as well as the deposition of Aβ proteins in the internal vessel walls, causing vascular occlusion; reduced blood; and, ultimately, retinal hypoxia [7].

Most studies that compared the thickness of the retinal nerve fiber layer (RNFL) and the ganglion cell-inner plexiform layer complex (GC-IPL) of AD patients with those of healthy controls agree that the former group demonstrates significant thinning in these areas compared to the latter [10,11,12]. In fact, although the overall mean RNFL is reduced in AD, the superior quadrant seems to be the area that is most noticeably affected [13,14]. However, a small number of studies have failed to confirm these findings [6,15].

Given that AD represents a stage of established dementia, meaning that by the time of diagnosis the patient’s health has already been severely affected, researchers have also focused on the prodromal phase of the disease, termed mild cognitive impairment (MCI). Zhang et al. found a significantly reduced parafoveal SCP VD in an MCI group compared to controls [8]. Additionally, according to a recent prospective study, there is a marked loss of VD in the DCP in MCI patients. This is in accordance with the findings of Jiang et al., who found a lower VD in the superonasal quadrant of the DCP [6]. In a study by Querques et al., a quantitative analysis of the retinal vessels of patients with MCI and AD was performed using a dynamic vessel analyzer and OCTA [16]. Although they noted a decrease in the reaction amplitude and the arterial dilation in the MCI group, the OCTA values did not show any significant variability between the groups. In fact, in a monozygotic twins preclinical AD study, researchers reported a higher VD in all areas in the Aβ-positive group, with no differences seen in the FAZ area [17]. Regarding RNFL thickness, most studies agree that this is significantly reduced in the MCI group when compared to controls [18,19,20]. Furthermore, a few studies have noted significant differences in the retinal thickness between AD and MCI [21,22,23].

The results of studies regarding the peripapillary vasculature appear to be incongruent. Although Lahme et al. demonstrated a reduced radial peripapillary capillary (RPC) VD in AD patients [24], Zabel et al. [9] found no statistically significant changes between the AD group and healthy controls. Finally, Zhang et al. [8] found no differences in the RPC values in the MCI group compared to controls. Thus, further research is needed in order to reliably pinpoint specific OCTA parameters that may be able to detect early forms of AD.

Choroidal thickness also seems to be a valuable biomarker for AD, as several studies have managed to demonstrate significant thinning in these patients compared to controls [25]. The pathophysiological mechanisms responsible for these findings are not completely clear yet. However, it seems that the accumulation of Aβ in the chοroid leads to inflammatory responses; complement activation; and, ultimately, to choroidal vascular angiopathy, in the same way as it occurs in AD brains [2].

The use of artificial intelligence and deep learning is constantly revolutionizing medicine and our understanding of certain diseases. A retinal OCTA segmentation database (ROSE) combined with an OCTA network has already been introduced and can provide the fractal dimension analysis of the retinal vasculature [26]. Despite being a novel method, the results appear to be promising, as researchers have already been able to detect significant changes between healthy controls and AD patients.

## 3. OCTA in Vascular Dementia

Vascular cognitive impairment and dementia (VCID), or vascular dementia (VaD), represents the second most common cause of cognitive impairment and a diagnostic challenge, in part due to the overlap with other dementia syndromes, including AD. The diagnosis of VaD, which is largely clinical, encompasses executive, visuospatial and/or memory dysfunction, among other cognitive aspects. There is a strong association with vasculopathy, including hypertension, hyperlipidemia and diabetes, which, through different mechanisms, ultimately lead to brain ischemia and degeneration [27]. White matter lesions or hyperintensities (WML/WMH), lacunar infarcts, microinfarcts, cerebral microbleeds and hemorrhages are the MRI hallmarks of this condition [28].

The retina is considered an extension of the cerebral tissue, and the study of its vasculature may reflect brain pathology. Therefore, OCTA is ideally suited to non-intrusively visualizing the functional microvascular changes which can be expected to be present in VaD. Indeed, it has been demonstrated that the flow density of the inner retinal layers might be a useful biomarker in differentiating vascular from degenerative dementia, as it correlates with the Fazekas scale but not with the presence of pathologic (Aβ, τ) proteins in the cerebrospinal fluid (CSF) [29,30]. A systematic review by Zhang et al. also revealed an association between WMHs and lower VD values, complementing the findings of Wang et al., who found the VD of the SCP to correlate with both WMHs and cognitive scores in patients with cerebral small-vessel disease (CSVD) [31,32]. Research in patients with subcortical vascular cognitive impairment (SVCI), a subtype of VaD, has shown lower capillary density (CD) values in the temporal RPC plexus as compared to healthy subjects. The researchers demonstrated that the CD values of the temporal and superior RPC quadrants were lower in the SVCI subgroup than in AD patients, a finding compatible with the potential use of this OCTA parameter as a differentiating biomarker. Furthermore, they noted a negative correlation between the RPC density and the CSVD score, an inherently important aspect of cognitive decline in VaD [28,33]. In a different study, patients with CSVD had lower VD values in both their temporal macular SCP and RPC plexuses versus healthy controls [32]. Of note, another recent study has shown VD to be significantly reduced in the DCP and RPC of healthy (i.e., cognitively normal) subjects with higher Fazekas scores as well, which highlights the potential of OCTA as an early biomarker [34]. The hypothesis that retinal microcirculation parameters might serve as biomarkers in VaD is further supported by the finding that lower vessel skeleton density (VSD)—a measure of perfused retina in OCTA images—is correlated with worse clinical (lower visuospatial and executive cognitive functions) and anatomical findings in patients with small-vessel disease (SVD) [29]. Specifically, visuospatial and executive cognitive functions and MRI findings related to cerebral perfusion and reactivity were found to be negatively affected. Cognitive function also appears to correlate with SCP density [32]. In one case report of a patient diagnosed with post-stroke VaD, a thinning of the choroid and electrophysiology consistent with outer retinal dysfunction was observed, which is argued to be in contrast to the inner retinal pathology reportedly associated with AD [35]. The underlying pathophysiological mechanism seems to be the expansion of the cerebral hypoperfusion to the choroidal circulation. However, additional research is required to supplement our knowledge of this case.

As VaD is inextricably linked to cerebrovascular disease (CVD), hereditary forms of CVD have fittingly been studied in the search for relevant retinal biomarkers. Cerebral autosomal dominant arteriopathy with subcortical infarcts and leukoencephalopathy (CADASIL) has indeed been found to be associated with lower VD in the DCP, affected RPC plexus and even choroidal thinning [30]. Similarly, another study suggests that the OCTA parameters of macular VD may be used as early biomarkers for Fabry disease, as these predict myocardial changes which might precede cognitive impairment [36].

An ambitious ongoing cohort study by Clancy et al. is performing an extensive examination of factors which affect the CSVD burden in patients with mild stroke. Among other tests, patients are being subjected to OCTA. The results of this study will hopefully further elucidate the relationship between retinal imaging and the risk of developing VaD, as well as solidifying its diagnosis [37].

## 4. Discussion

OCTA takes advantage of the ease of access to the retina through the clear media of the eye and produces fast, reproducible scans of structural as well as functional aspects of its microcirculation. Great interest has already been shown towards isolating useful biomarkers and, as this review has demonstrated, there is good reason to be optimistic about their potential routine use as such in the future. Existing data are much more abundant for AD in comparison to VaD, but at the same time they seem to be less conclusive with regard to certain aspects of the disease.

Patients with AD exhibit changes in macular VD, which has been reported to be diminished both as a whole as well as in either the SCP and/or DCP, depending on the study. VD changes might also be a feature of MCI. The inner retinal layers also appear to be consistently altered, with the GC-IPL being significantly thinned. There are conflicting findings regarding RNFL thickness and RPC density, which are, depending on the study, reported as being lower than seen in controls or unaffected. OCTA biomarkers that might help to consolidate the diagnosis of VaD are, for the most part, reflective of the vasculature rather than the structure of the retina. VD is generally found to be lower than controls, either in the SCP of CSVD patients or, in the case of hereditary CVD, in the DCP, or as a whole. The CD of the RPC also seems to be lower in SVCI (a VaD subtype) and hereditary CVD, and strongly correlated with the CSVD score. There may be a place for the further investigation of other less studied biomarkers, including VSD and flow density, which show promising results. RNFL thickness does not seem to be of particular importance as a biomarker in VaD. Structurally, choroidal thickness may be reduced in both AD and VaD.

Additional well-structured, cross-sectional and prospective studies are unquestionably required to provide concrete evidence of the applicability, sensitivity and specificity of each of these biomarkers in the differential diagnosis of degenerative and vascular dementia. However, some patterns seem to already be emerging: VD seems to be a common biomarker for both AD and VaD; inner layer, i.e., GC-IPL, thickness might represent a biomarker preferentially affected in AD, while, in contrast, the outer layer thickness as a whole justifies attention as a potential VaD biomarker; and RPC density is likely a biomarker specifically linked to VaD. Evidence on the relevance of RNFL in either form of dementia remains to be validated (Table 1). It might be the case that groups of biomarkers provide strong evidence in favor of one or the other form of dementia. As such, careful multivariate analysis would be advisable in future research in order to shed light on these relationships. One of the factors that needs to be considered in subsequent studies is ocular comorbidity, including macular degeneration and glaucoma—conditions which can interfere with the integrity of retinal tissue and are not uncommon in the age group usually suffering from cognitive impairment—as well as the effects of normal ageing in the retina and software artifacts.

## 5. Conclusions

OCT and OCTA are imaging modalities that are used in the everyday practice of all ophthalmologists and have become integral parts of the study of the retina and cho- roid. So far, neurologists are familiar with the use of OCT in neurological conditions such as multiple sclerosis. Thus, the aim of this review is to draw attention towards another possible application of this expanding technology. The available studies are limited and data suggest that certain OCT/OCTA findings may overlap in AD and VaD. However, it seems that some OCTA markers present promising targets for further research in this field. This review aims to summarize the current available data and may serve as a reference point for future research, such as the study of OCTA as a monitoring tool for longitudinal changes in various neurodegenerative diseases.

## Figures and Tables

**Table 1:** Suggested Alzheimer’s disease and vascular dementia OCT/OCTA biomarkers.

	Alzheimer’s Disease	Vascular Dementia
RPC	Lowered or unaffected	Lowered
RNFL thickness	Lowered or unaffected	Not of particular importance
Vessel Density	Decreased in SCP and/or DCP	Decreased in SCP and/or DCP
FAZ	Enlarged	No available data
Inner retinal layers	Thinning	No available data
Outer retinal layers	No available data	Thinning
Choroid	Thinning	Thinning

RPC: radial peripapillary capillary; RNFL: retinal nerve fiber layer; SCP: superficial capillary plexus; DCP: deep capillary plexus; FAZ: foveal avascular zone.

## Data Availability

Not applicable.
